# The Soft Palate

**Published:** 1872-04

**Authors:** 


### THE SOFT PALATE,

Or uvula, was formerly supposed to be a mere excrescence, having no appreciable uses, except to be a great bother sometimes to that unfortunate class of persons who, having nothing to do and plenty of time to do it in, spent a considerable portion of their existence in looking out for "symptoms;" the pernicious habit being cultivated by reading long articles in the newspapers about catarrh and bronchitis and consumption, until every hack or hem of the throat, every sniffle of the nose, every trifling effort at a cough, is considered an omen of evil things, a knell of death, if the advertiser was not immediately consulted, at the rate of fifty dollars an hour, with a view to getting the matter rectified by poking a young ramrod down the throat, with a bit of sponge fastened at the end, soaked in aquafortis or diluted molasses.

Some fifty years ago the great Doctor Physic, of Philadelphia, was consulted by a gentleman who had travelled the world over to find a cure for a troublesome cough, and an everlasting disposition to swallow his head down into his stomach. No physician at home or abroad had ever done him the least particle of good, except to relieve him of the trouble of carrying so much gold about his person. For didn't it "stand to reason" that if a man was sick and weak and debilitated, the less he had to carry the better? His body was worn to a skeleton; his countenance looked like the tomb of the dead — so utterly woe-begone that it spread a funereal gloom over any company the moment the room was entered. "Open your mouth," said the doctor. In an instant a great cavern was exposed, dark, gloomy, and peculiar. "Steady now, hold still until I can feel it." The next instant a miniature snake was exhibited to the astonished patient, in the shape of a soft, slender piece of flesh, the like of which the man had never seen before, and he was all curiosity to know what it was. The physician went on to explain that from some cause the soft palate had become too long; and between its getting up in the nose and down into the windpipe or across into the swallow, it had been keeping the poor man all this time in hot water; but that it was all over now: the seat of the trouble had been discovered, and he would get steadily well; and it was so. The soft palate was too long. The incident has been repeated once a year ever since, in every medical college in the United States, with a practical effect, which may reach the reader; for countless numbers of young doctors go off with their diplomas, having the incident so strongly impressed on their minds that they are determined that every man, woman, and child who comes with any kind of tickling in the throat, or unavailing swallowing, or inconvenient hacks or hems or clearing of the throat, shall have the uvula sliced off instantly, in the hope of a like miraculous result, to the end of getting a great name for sagacity, skill, and acuteness. It is not likely that a similar result would follow such an operation more than once in a million of times.

It is not remembered at this writing that the case has been repeated once since Physic's day.

A few years ago a physician was established in this city, who whacked off about ten thousand uvulas, at a charge of ten dollars each, when at last he performed his peculiar manipulation once too often, and the patient died next day of a throat operation. It broke the doctor's heart, and he died too. He never got over the mortification.

No one ought to allow the uvula or soft palate to be clipped off, until it is considered indispensably necessary by a surgeon or physician of repute and acknowledged ability; because its offices are important and various.

1st. It admonishes the little bridge which spans the top of the windpipe that something is about to pass over it; unless that is done the food, instead of going over this bridge into the throat, down into the stomach, would fall into the windpipe, and the person would be strangled to death. And when it is remembered that every morsel eaten, and every drop swallowed, has to pass over this bridge, and that the uvula gives warning, its integrity is a matter of considerable importance.

2d. In the act of swallowing the uvula swings back and upwards as on a hinge, and perfectly closes the passage from the mouth to the nose, every time a person swallows; otherwise, in the attempt to swallow, the matters would be forced upwards and come out through the nose, as happens sometimes, when by any means the parts are not properly adjusted, as in partial strangulation.

3d. The uvula aids in giving guttural sounds to the voice, as well as in giving the highest notes in singing.

It is not at all likely that more than one person in a million is benefited by losing the uvula, beyond the day or two which is required for getting rid of the inflammation induced by the operation. Sometimes it swells from a cold; at others it becomes relaxed and falls down; all that is needed in such cases is a gargle made by a walnut-sized bit of alum in a quart of water, used three or four times a day.

